# Hypochlorite-induced oxidative stress elevates the capability of HDL in promoting breast cancer metastasis

**DOI:** 10.1186/1479-5876-10-65

**Published:** 2012-03-30

**Authors:** Bing Pan, Hui Ren, Xiaofeng Lv, Yangyu Zhao, Baoqi Yu, Yubin He, Yijing Ma, Chenguang Niu, Jinge Kong, Fangzhu Yu, Wen-bing Sun, Youyi Zhang, Belinda Willard, Lemin Zheng

**Affiliations:** 1 The Institute of Cardiovascular Sciences and Institute of Systems Biomedicine, School of Basic Medical Sciences, and Key Laboratory of Molecular Cardiovascular Sciences, Ministry of Education, Key Laboratory of Cardiovascular Molecular Biology and Regulatory Peptides, Ministry of Health, Peking University Health Science Center, Beijing 100191, China; 2 The Department of Cardiovascular Medicine and the Department of endocrinology, the Military General Hospital of Beijing, Beijing 100700, China; 3 The Department of Obstetrics and Gynecology and the Institute of Vascular Medicine, Peking University Third Hospital, Beijing 100191, China; 4 Department of Hepatobiliary Surgery, West Campus, Beijing Chao-yang Hospital, Capital Medical University, Beijing 100043, China; 5 Proteomics Laboratory, Cleveland Clinic, Cleveland, OH 44195, USA; 6 Institute of Cardiovascular Sciences, Peking University Health Science Center, 38 Xueyuan Road, Haidian District, Beijing 100191, China

**Keywords:** Breast cancer, Oxidative stress, Metastasis, High-density lipoprotein

## Abstract

**Background:**

Previous studies suggest that oxidative stress plays an important role in the development of breast cancer. There is a significant inverse relationship between HDL and the risk and mortality of breast cancer. However, it is well known that under conditions of oxidative stress, such as breast cancer, HDL can be oxidatively modifiedand these modifications may have an effect on the functions of HDL. The purpose of this study is to determine the different effects of normal and oxidized (caused by hypochlorite-induced oxidative stress) HDL on breast cancer cell metastasis.

**Methods:**

Human breast cancer cell lines were treated with normal and hypochlorite-oxidized HDL, and then cell metastasis potency *in vivo *and the abilities of migration, invasion, adhesion to HUVEC and ECM *in vitro *were examined. Integrin expression and PKC activity were evaluated, and PKC inhibitor and PKC siRNA was applied.

**Results:**

We found hypochlorite-oxidized HDL dramatically promotes breast cancer cell pulmonary metastasis (133.4% increase at *P *< 0.0 l for MDA-MB-231 by mammary fat pad injection; 164.3% increase at *P *< 0.01 for MCF7 by tail vein injection) and hepatic metastasis (420% increase at *P *< 0.0 l for MDA-MB-231 by mammary fat pad injection; 1840% fold increase at *P *< 0.001 for MCF7 by tail vein injection) in nude mice, and stimulates higher cell invasion (85.1% increase at *P *< 0.00 l for MDA-MB-231; 88.8% increase at *P *< 0.00 l for MCF7;), TC-HUVEC adhesion (43.4% increase at *P *< 0.00 l for MDA-MB-231; 35.2% increase at *P *< 0.00 l for MCF7), and TC-ECM attachment (41.0% increase at *P *< 0.00 l for MDA-MB-231; 26.7% increase at *P *< 0.05 for MCF7) *in vitro *compared with normal HDL. The data also shows that the PKC pathway is involved in the abnormal actions of hypochlorite-oxidized HDL.

**Conclusions:**

Our study demonstrated that HDL under hypochlorite-induced oxidative stress stimulates breast cancer cell migration, invasion, adhesion to HUVEC and ECM, thereby promoting metastasis of breast cancer. These results suggest that HDL-based treatments should be considered for treatment of breast cancer patients.

## Background

Breast cancer is the most common malignant neoplasm among women worldwide, and is one of the leading causes of cancer-related death [1]. Annually, about one million women are newly diagnosed with breast cancer and 400,000 patients die from the disease [2]. The etiology of breast cancer is multifactorial. It is suggested that oxidative stress which is responsible for protein, lipid and DNA damage plays an important role in the development and progression of breast cancer [3]. Hypochlorite is an important ROS, which can be released by activated white blood cells in different diseases during pro-inflammatory states [4]. However, the role of oxidative stress in the progression of breast cancer is not fully understood.

Metastasis of breast cancer is the major cause of tumor-related morbidity and mortality [5,6]. It is a multistep process in which tumor cells migrate and invade into adjoining cells and tissues, attach to subendothelial extracellular matrix (ECM) and endothelial cells of blood vessels, and proliferate at other sites to establish secondary tumors [7]. Many of these steps involve integrins, a glycoprotein family which consist of an α subunit noncovalently linked to a β subunit [8]. Integrins have been implicated in a wide variety of cellular functions, including cell migration, invasion, adhesion, proliferation and survival [8,9]. Another factor that is has been shown to be involved in the signaling pathways that regulate tumor cell proliferation, differentiation, apoptosis, motility and adhesion is the serine/threonine kinase protein kinase C (PKC) [10,11]. Interestingly, PKC activity has been shown to be important in the regulation of integrins expression, localization [12,13] and activity [14].

High-density lipoprotein (HDL), a heterogeneous class of lipid- and protein- containing plasma particles, is commonly known for its atheroprotective functions [15,16]. Furthermore, it has been reported that HDL levels are inversely related to the incidence of various cancer, including breast cancer [17-20]. However, several studies have shown that the modification of HDL can impair its atheroprotective functions [21,22]. For instance, we previously found that HDL of patients with type 2 diabetes mellitus elevates the capability of HDL to promote metastasis of breast cancer *in vivo *[23], and stimulate cell proliferation, migration, invasion, adhesion to HUVEC and attachment to EC *in vitro *[24]. Moreover, oxidative stress could lead to lipid peroxidation and protein modification, and such changes may significantly affect the functions of HDL.

In the present study, we investigated how hypochlorite-oxidized HDL (which was produced *in vitro *to mimic the alteration of HDL under oxidative stress) differentially affects the metastatic progression of human breast cancer cells *in vivo *compared with normal HDL. In order to more fully understand the mechanisms of these actions, both the levels of integrins and the PKC pathway were measured. Our findings may contribute to the understanding of the relationship between oxidative stress and breast cancer, and promote the need for HDL-altering therapy in breast cancer patients.

## Methods

### Animals

4 week old female BALB/c nude mice were obtained from the animal house, Academy of Military Medical Sciences, China. Throughout the experiments, mice were maintained with free access to pellet food and water. All experimental procedures were approved by the Ethics Committee of Animal Research, Peking University Health Science Center, and the investigation conformed to the Guide for the Care and Use of Laboratory Animals published by the US National Institutes of Health (NIH Publication No. 85-23, revised 1996).

### Cell lines and cell culture

The hormone-independent MDA-MB-231 and hormone-dependent MCF7 human breast cancer cell lines were from Cell Resource Center, Institute of Basic Medical Sciences, Chinese Academy of Medical Sciences, and cultured in Dulbecco's modified Eagle's medium (DMEM; GIBCO, UK) containing 10% fetal bovine serum (FBS; GIBCO, UK) in a humidified incubator at 37°C with an atmosphere of 5% CO_2_. Human umbilical vein endothelial cells (HUVEC) were isolated by collagenase digestion of umbilical veins from fresh cords [25]. The cells were plated on gelatin-coated culture dishes in Endothelial Cell Medium (ECM; Sciencell, USA) consisting of 500 ml of basal medium, 25 ml of fetal bovine serum, 5 ml of endothelial cell growth supplement and 5 ml of penicillin/streptomycin solution and then cultured in a humidified incubator at 37°C with an atmosphere of 5% CO_2_. HUVEC were used at passages 2-5.

### Isolation and hypochlorite-oxidation of HDL

Fresh, fasting plasma was separated by centrifugation from peripheral blood obtained from healthy subjects (referred to as N-HDL). The study protocol was approved by the local ethics committee. HDL was isolated from fresh plasma by ultracentrifugation, dialyzed against 3 × 1 L of endotoxin-free PBS (pH = 7.4), sterilized with 0.22 μm filter, stored in sealed tubes at 4°C in dark and used within 2 months [26]. Hypochlorite-oxidized HDL (referred to as H-HDL) performed as described previously [27]. Briefly, the concentration of NaOCl was calculated using a molar absorption coefficient of 350 M^-1 ^cm^-1 ^at 292 nm. HDL was incubated with NaOCl at 37°C for 1 hour and then at 4°C overnight (oxidant:protein molar ratios = 30:1). Then samples were dialyzed overnight at 4°C against standard buffer (10 mM Na phosphate, 100 μM DTPA, pH 7.4).

### MTT assay

Cells (3 × 10^3^/well) were seeded in 96-well plates and were subsequently incubated for 12 hours to allow attachment. Then cells were incubated with HDL (at 100 μg/ml apoA-I) for 12, 24, 48 and 72 hours respectively. 4 hours before the end of incubation, 20 μl of 5 mg/ml MTT (3-[4,5-dimethylthiazol-2-yl]-2,5-diphenyl tetrazolium bromide; Ameresco, USA) was added to each well. At the end of incubation, the medium was removed and 150 μl/well DMSO was added to solubilize the formazan. Finally, the optical density of the soluble formazan was read at 570 nm with a plate reader (model 550, BioRad, USA). The average values were determined from quadruplicate readings.

### Wound healing migration assay

Cells were seeded onto 24-well plates and cultured till to form monolayers, then wounded by manual scraping with a 200 μl-micropipette tip. Cells were then incubated with medium containing 1%FBS alone or together with indicated concentrations of HDL for 24 hours (for MDA-MB-231 cells) or 48 hours (for MCF7 cells). Cells were fixed with methanol, stained with hematoxylin and eosin, and photographed with an inverted microscope (Nikon, Japan). Cell migration was quantified by counting the number of cells that migrated into the gap in 10 random high power (100×) fields.

### Transwell migration assay

Quantitative cell migration assay was performed using a modified Boyden chamber (Minicell, Millipore, USA) with 8.0-μm pore polycarbonate filter inserts in 24-well plates. The lower chamber was filled with 0.6 ml of DMEM containing 10% FBS. Cells (1 × 10^5 ^cells/well) in serum-free medium were plated into the upper chamber with DMEM alone or with indicated concentrations of HDL. After 6 hours (for MDA-MB-231 cells) or 18 hours (for MCF7 cells) incubation, all non-migrated cells were removed from the upper face of the transwell membrane by a cotton swab, and the migrated cells were fixed with methanol, stained with hematoxylin and eosin, and photographed under an inverted microscope (Nikon, Japan). Migration was quantified by counting the number of stained cells from 10 random high power (100×) fields.

### Transwell invasion assay

Quantitative cell invasion assay was performed using a modified Boyden chamber (Minicell, Millipore, USA) with 8.0-μm pore polycarbonate filter inserts in 24-well plates. The lower face of the polycarbonate filter (transwell insert) was coated with 20 μl Matrigel (BD Bioscience, Germany) for one hour at 37°C and then air dry. The lower chamber was filled with 0.6 ml of DMEM containing 10% FBS. Cells (1 × 10^5 ^cells/well) in serum-free medium were plated into the upper chamber with or without indicated concentrations of HDL. After 18 hours (for MDA-MB-231 cells) or 24 hours (for MCF7 cells) incubation, all non-invading cells were removed from the upper face of the transwell membrane by a cotton swab, and the invaded cells were fixed with 10% formalin, stained with hematoxylin and eosin, and photographed under an inverted microscope (Nikon, Japan). Invasion was quantified by counting the number of stained cells from 10 random high power (100×) fields.

### TC-HUVEC adhesion assay

TC-HUVEC adhesion was measured using rose Bengal stain as previously described method [28]. Briefly, HUVEC were seeded onto 96-well plates at a density of 5 × 10^4 ^cells/well. Then tumor cells (pretreated with or without HDL for 6 hours) were plated (5 × 10^4 ^cells/well) and incubated for 30 minutes at 37°C. Then unattached cells were gently washed twice with 10% FBS-containing DMEM, and 100 μl of 0.25% rose Bengal (Sigma, USA) was added. After 5 minutes, cells were gently washed twice, and then 200 μl ethanol/PBS (1:1) was added to each well. After 30 minutes, the absorbance at 570 nm was recorded. Five parallel wells were set up for each group.

### TC-ECM adhesion assay

Cell adhesion was measured using MTT assay as previously described method [29]. Briefly, 96-well flat bottom plates were coated with 2 μg/well basement membrane matrix (Matrigel, BD, Germany) for a hour at 37°C then blocked with 2% BSA for 2 hours at 37°C followed by washing twice. Tumor cells seeded on 12-well plate were treated with serum-free DMEM alone or HDL for 6 hours. After detachment with trypsin, the cells (1 × 10^4^/well) were plated on ECM-precoated 96-well plates and then washed twice with PBS to remove non-adherent cells after 45 minutes. MTT colorimetric assays at 490 nm were employed to measure the absorbance of adhesive cells. Four parallel wells were set up for each group.

### Determination of cell surface expression of adhesion molecules by enzyme immunoassay

Cells in 96-well plates were grown till 70% confluency, then treated with normal or oxidized HDL in serum-free medium for 6 hours at 37°C. Cells were washed with PBS and fixed with methanol for 5 minutes and then washed with PBS 3 times and blocked with 2% BSA for 2 hours at 37°C. Cell surface expression of adhesion molecules were determined by primary binding with specific monoclonal antibodies for integrin β1, integrin β2, integrin β3, integrin αv (1:200; Abcam, Hong Kong) followed by secondary binding with a horseradish peroxidase-conjugated antibody (1:3000; Boster, China) as described previously [30]. Quantification was performed by determination of colorimetric conversion at an optical density at 450 nm of 3,3,5,5-tetramethylbenzidine using TMB peroxidase EIA substrate kit (Bio-Med innovation, China).

### PepTag assay for nonradioactive detection of PKC activity

The PKC activity in the MDA-MB-231 and MCF7 cell lysates was determined by non-radioactive detection kit of protein kinase (PepTag Corporation, USA), following the manufacturer's instructions. Briefly, PKC in the HDL-treated cell lysates was separated by column of DEAE cellulose and was incubated with the brightly colored, fluorescent peptide substrates. Then phosphorylation by PKC of its specific substrate alters the peptide's net charge from +1 to -1, which allows the phosphorylated and non-phosphorylated versions of the substrate to be rapidly separated on an agarose gel. For quantification, the blots were analyzed using Gel-Pro analyzer 3.0 software and quantification was based on the IOD (Integrated Optical Density) value of each band.

### siRNA knockdown of PKC

MDA-MB-231 cells were plated onto 6-well plates and allowed to grow to sub-confluent. Cells were transiently transfected with negative control siRNA or PKC siRNA (Santa Cruz, America) by lipofectamine RNAi MIX reagent (Invitrogen, America) in OPTI-MEM medium (Gibco, America) for 6 h, and then incubated in ECM with 5% FBS. Cells were prepared to be used for further experiments.

### Tail vein metastasis model

MCF7 cells were pretreated with normal and oxidized HDL for 24 hours. To produce experimental metastasis, BALB/c nude mice (6 mice were set for each group) were injected intravenously with MCF7 cells (4 × 10^5^) in 0.2 ml DMEM via tail veins. After 20 days, the mice were sacrificed, their lungs and livers were resected and photographs were taken. Numbers of metastatic nodules on the surface of the lungs and livers were counted.

### Mammary fat pad spontaneous metastasis model

MDA-MB-231 cells were pretreated normal and oxidized HDL for 24 hours and then 2 × 10^6 ^cells suspended in DMEM were injected into the mammary fat pad. BALB/c nude mice (6 mice were set for each group) were received normal or oxidized HDL injection through vein tail every three days. After 40 days, the mice were sacrificed, their lungs and livers were resected and photographs were taken. Numbers of metastatic nodules on the surface of the lungs and livers were counted.

### Histology

Tissues were fixed in buffered formalin for 24-48 h. After washing in fresh PBS, fixed tissues were processed and embedded in paraffin. Sections (5 milli micron) were collected on microscope slides, deparaffinized and stained with H&E. The images were captured at 10x magnification.

### Statistical analysis

The results of multiple observations are presented as the mean ± SEM and as a representative result of three different separate experiments unless otherwise stated. Data were analyzed using Student's t test and ANOVA test and values were considered significant at *P *< 0.05.

## Results

### Hypochlorite-modified HDL is significantly more effective in stimulating breast cancer cell proliferation, migration and invasion

**Figure 1:**
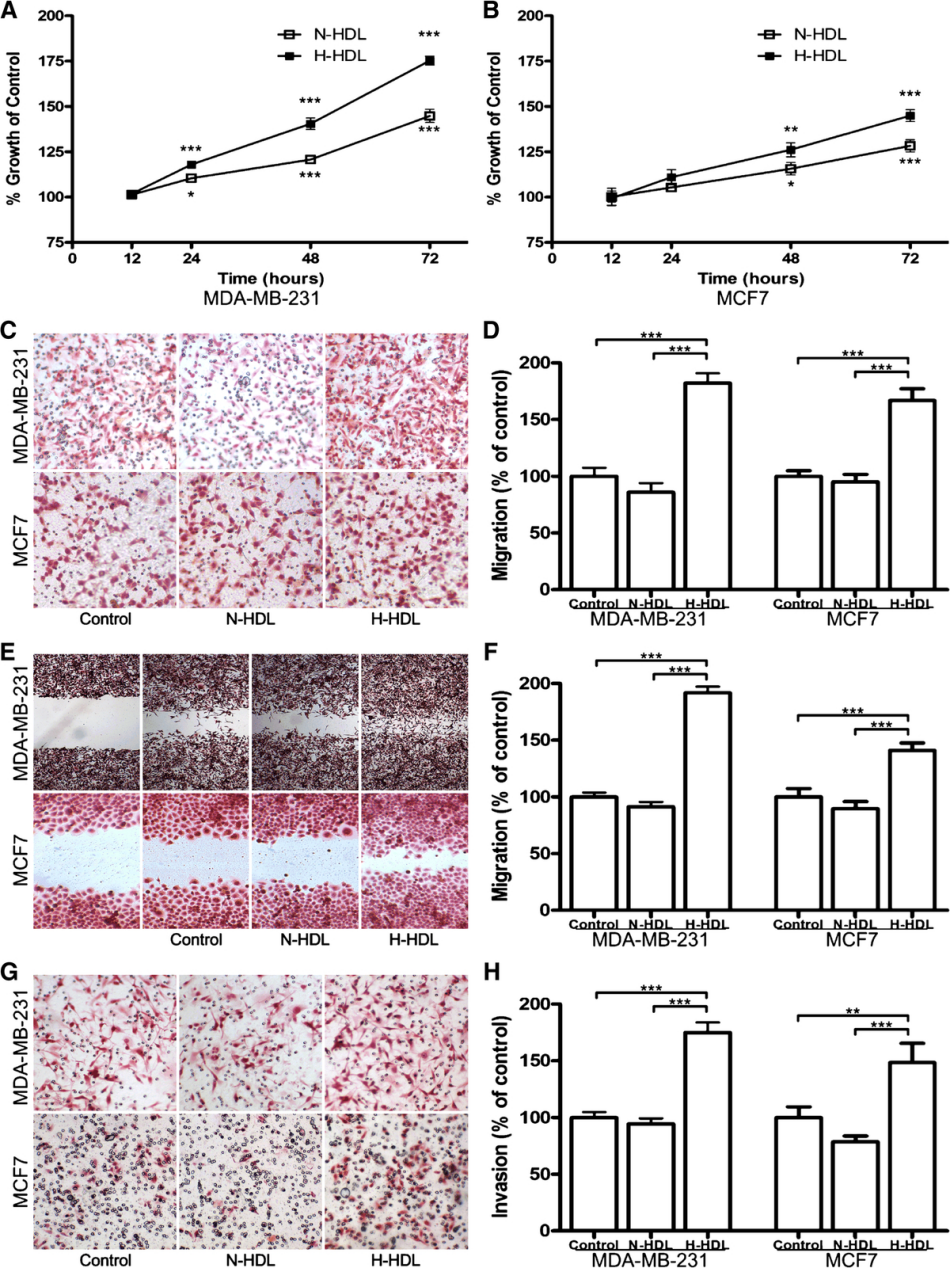
**H-HDL is more effective in stimulating MDA-MB-231 and MCF7 cell proliferation, migration and invasion**. MDA-MB-231 **(a) **and MCF7 **(b) **were treated with N-HDL and H-HDL respectively for 12, 24, 48, 72 hours at 100 μg/ml apoA-I. Relative cell proliferation was measured by MTT assay and expressed as percentage of HDL treated cells in comparison to control. (*, *P *< 0.05; **, *P *< 0.01; ***, *P *< 0.001; one-way ANOVA). **(c) **MDA-MB-231 and MCF7 cells were incubated with N-HDL and H-HDL respectively at 100 μg/ml apoA-I in upper chamber for indicated time. Transwell assay was used to measure cell migration and pictures were shown in high power (100×). **(d) **Migrated cells were counted and the results were expressed as percentage of HDL treated cells in comparison to control. (***, *P *< 0.001; one-way ANOVA). **(e) **Monolayer MDA-MB-231 and MCF7 cells were wounded by manual scraping and incubated with N-HDL and H-HDL respectively at 100 μg/ml apoA-I for indicated time and then photographed in high power (100×). **(f) **Cells migrated into the gaps were counted and the results were expressed as percentage of HDL treated cells in comparison to control. (***, *P *< 0.001; one-way ANOVA). **(g) **MDA-MB-231 and MCF7 cells were treated with N-HDL and H-HDL respectively in upper chamber for indicated time at 100 μg/ml apoA-I. Transwell assay was used to measure cell invasion and pictures were shown in high power (100×). **(h) **Invasive cells were counted and the results were expressed as percentage of HDL treated cells in comparison to control. (**, *P *< 0.01; ***, *P *< 0.001; one-way ANOVA).

We first determined the effects of N-HDL and H-HDL on cell proliferation in two different breast cancer cell lines. MDA-MB-231 and MCF7 cells were incubated with N-HDL and H-HDL for 12, 24, 48 and 72 hour at 100 μg/ml apoA-I. It was found that both N-HDL and H-HDL stimulated MDA-MB-231 and MCF7 cell proliferation in a time dependent manner (*P *< 0.00 l; Figure 1A, B). Of note, H-HDL presented to be a more powerful stimulant of proliferation for both MDA-MB-231 (Figure 1A) and MCF7 cells (Figure 1B). For MDA-MB-231 cells, cell proliferation induced by H-HDL increased by 6.9% at 24 hours (*P *< 0.05), 16.4% at 48 hours (*P *< 0.0 l) and 21.0% at 72 hours (*P *< 0.00 l) compared with N-HDL. For MCF7 cells, cell proliferation induced by H-HDL increased by 14.5% at 72 hours (*P *< 0.05) compared with N-HDL.

We further investigated the effects of N-HDL and H-HDL on the cell migration and invasion of MDA-MB-231 and MCF7 cell lines. It was shown that H-HDL promotes MDA-MB-231 and MCF7 cell migration and invasion while N-HDL was inhibitory. For MDA-MB-231 cells, the migration induced by H-HDL increased by 111.9% and 82.0% in the transwell migration assay and by 110.1% and 91.7% in the wound healing assay compared with N-HDL and control respectively (both at *P *< 0.00 l; Figure 1C, D, E, F). In addition, the invasive MDA-MB-231 cells induced by H-HDL increased by 85.1% and 74.8% in the transwell invasion assay compared with N-HDL and control respectively (both at *P *< 0.00 l; Figure 1G, H). For MCF7 cells, the migration induced by H-HDL increased by 75.3% and 66.8% in transwell migration assay and by 57.5% and 41.1% in wound healing assay compared with N-HDL and control respectively (both at *P *< 0.00 l; Figure 1C, D, E, F). In addition, the invasive MDA-MB-231 cells induced by H-HDL increased by 88.8% and 48.4% in transwell invasion assay compared with N-HDL and control respectively (*P *< 0.00 l for N-HDL and *P *< 0.0 l for control; Figure 1G, H).

### Breast cancer cells pretreated with hypochlorite-modified HDL have increased capacities of adhesion to HUVEC and attachment to ECM

**Figure 2:**
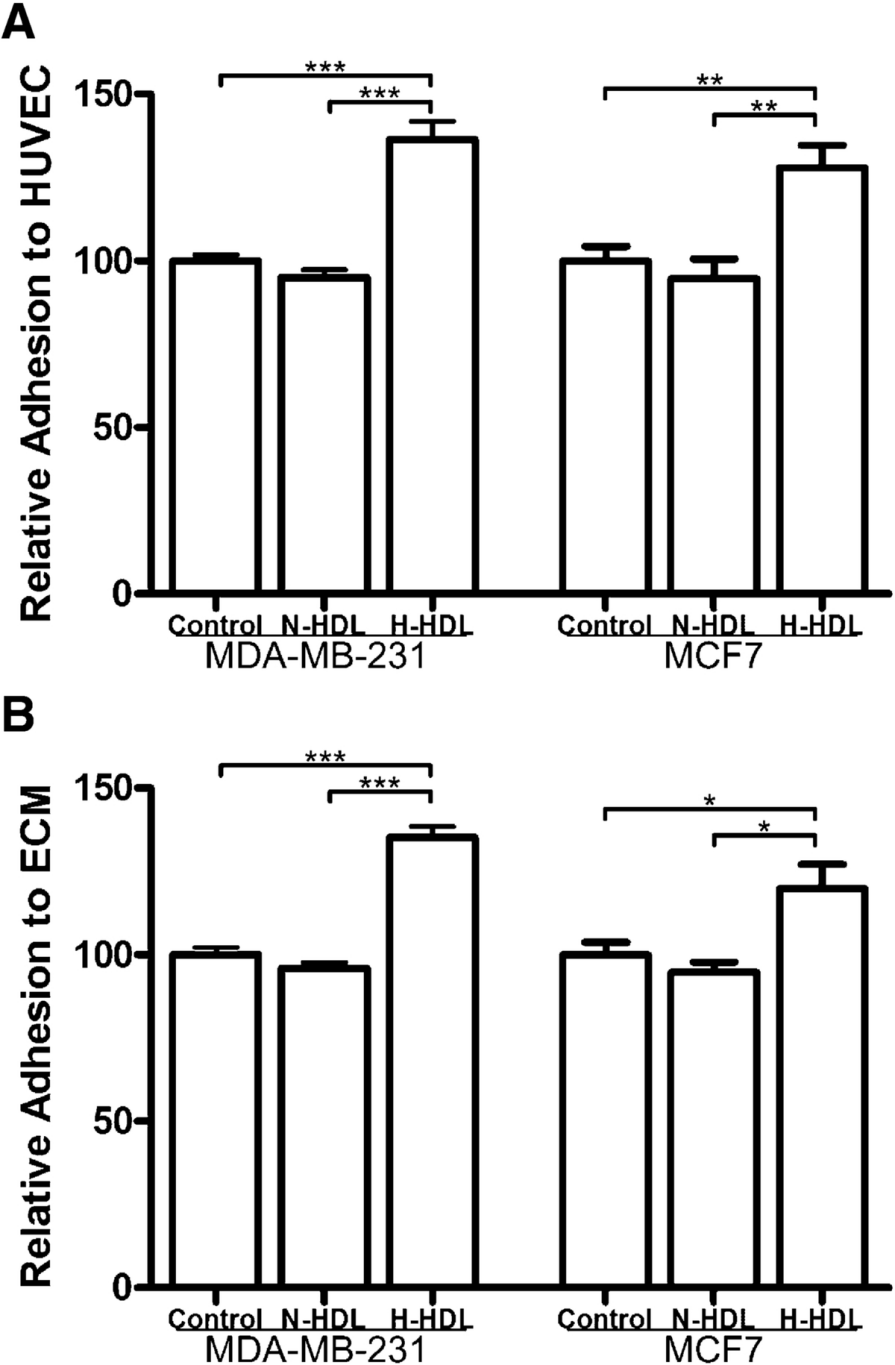
**H-HDL promotes more MDA-MB-231 and MCF7 cell adhesion to HUVEC and attachment to ECM**. **(a) **MDA-MB-231 and MCF7 cells were pretreated with N-HDL and H-HDL at 100 μg/ml apoA-I for 6 hours and then seeded onto HUVEC-coated 96-well plate for 30 minutes. Relative cell adhesion was determined by Rose Bengal assay and expressed as percentage of HDL treated cells in comparison to control. Data is expressed as mean ± SEM with five parallel wells. (**, *P *< 0.01; ***, *P *< 0.001; one-way ANOVA). **(b) **MDA-MB-231 and MCF7 cells were pretreated with N-HDL and H-HDL at 100 μg/ml apoA-I for 6 hours and then plated onto matrigel-coated 96-well plate for 30 minutes. Relative cell adhesion was determined by MTT assay and expressed as percentage of HDL treated cells in comparison to control. Data is expressed as mean ± SEM with four parallel wells. (*, *P *< 0.05; ***, *P *< 0.001; one-way ANOVA).

The effects of N-HDL and H-HDL on MDA-MB-231 and MCF7 cell adhesion to HUVEC and attachment to ECM was tested. It was shown that the adhesion of MDA-MB-231 and MCF7 cells to both HUVEC (Figure 2A) and ECM (Figure 2B) pretreated with H-HDL was markedly increased compared to cells pretreated with N-HDL and control. In the TC-HUVEC adhesion assay, the adhesion of MDA-MB-231 cells pretreated with H-HDL increased by 43.4% and 36.4% as compared with N-HDL and control respectively (both at *P *< 0.001; Figure 2A). Similarly, the adhesion of MCF7 cells pretreated with H-HDL increased by 35.2% and 28.0% as compared with N-HDL and control respectively (both at *P *< 0.00 l; Figure 2A). In the TC-ECM adhesion assay, the attachment of MDA-MB-231 cells pretreated with H-HDL increased by 41.0% and 35.2% as compared with N-HDL and control respectively (both at *P *< 0.001; Figure 2B). Similarly, the attachment of MCF7 cells pretreated with H-HDL increased by 26.7% and 19.9% as compared with N-HDL and control respectively (both at *P *< 0.05; Figure 2B).

**Figure 3:**
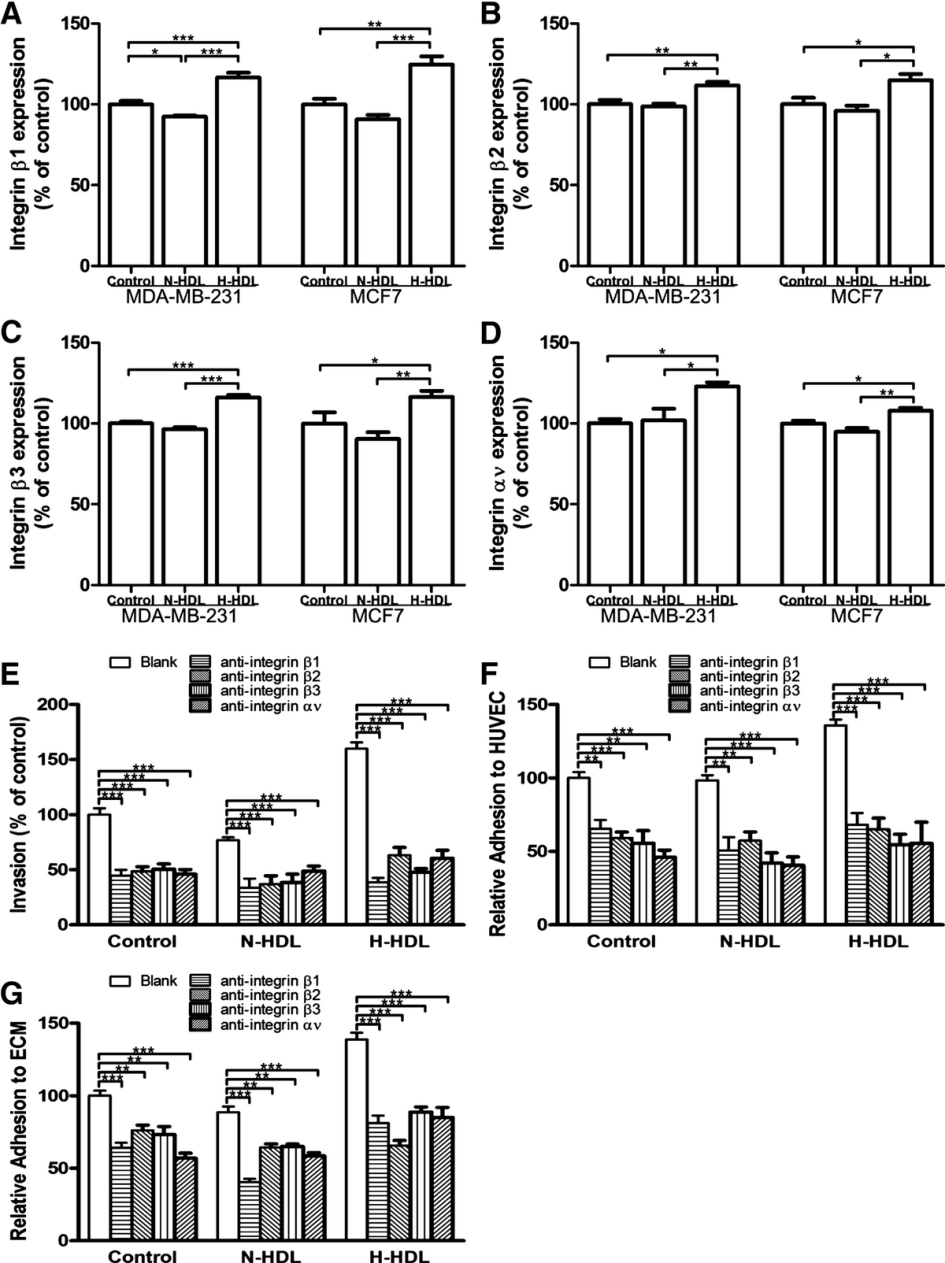
**Intergrins are involved in the increased invasion and adhesion of breast cancer cells induced by H-HDL**.MDA-MB-231 and MCF7 cells were treated with N-HDL and H-HDL at 100 μg/ml apoA-I for 6 hours. Integrin β1 **(a)**, integrin β2 **(b)**, integrin β3 **(c) **and integrin αv **(d) **levels on the cell surface were measured by cell ELISA with five parallel wells. Results were expressed as percentage of HDL treated cells in comparison to control and data is expressed as mean ± SEM. (*, *P *< 0.05; **, *P *< 0.01; ***, *P *< 0.001 by one-way ANOVA). **(e) **MDA-MB-231 cells were incubated with N-HDL and H-HDL at 100 μg/ml apoA-I along with no antibody, negative control antibody, or anti-integrin (β1, β2, β3, αv) antibodies respectively in upper chamber for 18 hours. (***, *P *< 0.001; one-way ANOVA). MDA-MB-231 cells were pretreated with N-HDL and H-HDL at 100 μg/ml apoA-I for 6 hours and then incubated with no antibody, negative control antibody, or anti-integrin (β1, β2, β3, αv) antibodies for 30 minutes at 37°C before the adhesion to HUVEC **(f) **and attachment to ECM **(g)**. Antibody-blocked groups were compared with control (antibody-untreated groups). Data is expressed as mean ± SEM with five parallel wells. (**, *P *< 0.01; ***, *P *< 0.001; one-way ANOVA).

**Integrins are involved in hypochlorite-modified HDL-induced invasion and adhesion of breast cancer cells**. We previously found integrin β1, integrin β2, integrin β3 and integrin αν play an important role in the in the adhesion of MDA-MB-231 and MCF7 cells to HUVEC and ECM by using blocking antibodies to intergrins [23]. In the present study, it was found that H-HDL induced elevated expression levels of integrin β1 (Figure 3A), integrin β2 (Figure 3B), integrin β3 (Figure 3C) and integrin αv (Figure 3D) compared with N-HDL on both the MDA-MB-231 and MCF7 cell surface. Specifically, for MDA-MB-231 cells, integrin β1 was increased by 26.4% and 16.6% respectively (both at *P *< 0.001; Figure 3A); integrin β2 was increased by 13.2% and 11.7% respectively (both at *P *< 0.01; Figure 3B); integrin β3 was increased by 20.3% and 16.0% respectively (both at *P *< 0.001; Figure 3C); integrin αv was increased by 20.6% and 22.9% respectively (both at *P *< 0.05; Figure 3D). For MCF7 cells, integrin β1 was increased by 37.4% and 24.6% respectively (*P *< 0.001 for N-HDL, *P *< 0.01 for control; Figure 3A); integrin β2 was increased by 19.6% and 14.8% respectively (both at *P *< 0.05; Figure 3B); integrin β3 was increased by 28.8% and 16.4% respectively (*P *< 0.01 for N-HDL, *P *< 0.05 for control; Figure 3C); integrin αv was increased by 13.8% and 7.9% respectively (*P *< 0.01 for N-HDL, *P *< 0.05 for control; Figure 3D). Furthermore, after treatment with blocking antibodies to integrins, MDA-MB-231 cells invasion (Figure 3E), adhesion to HUVEC (Figure 3F), and attachment to ECM (Figure 3G) was dramatically reduced in all the control, N-HDL and H-HDL groups.

### PKC inhibitor significantly reduces hypochlorite-modified HDL-induced migration, invasion and adhesion of MDA-MB-231 and MCF7 cells

**Figure 4:**
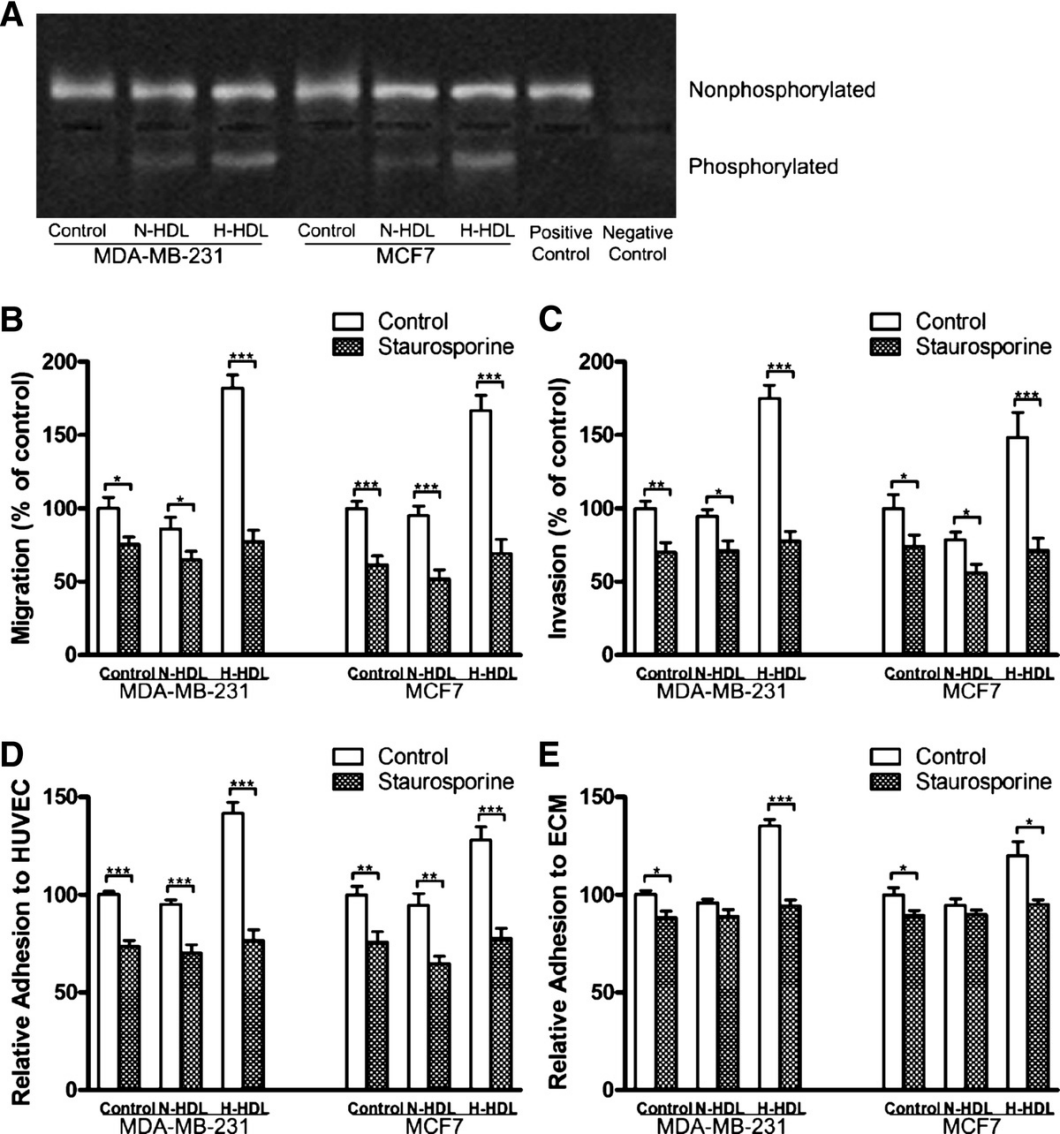
**H-HDL stimulates MDA-MB-231 and MCF7 cell migration, invasion and adhesion involving PKC pathway**. **(a)**MDA-MB-231 and MCF7 cells were treated with N-HDL and H-HDL for 30 minutes at 100 μg/ml apoA-I, and then the cell lysates were subjected to the PepTag nonradioactive PKC assay. **(b, c) **MDA-MB-231 and MCF7 cells were preincubated with 5 nM staurosporine, a PKC inhibitor along with N-HDL and H-HDL at 100 μg/ml apoA-I respectively for indicated hours (6 hours for MDA-MB-231 and 18 hours for MCF7 in transwell migration assay; 18 hours for MDA-MB-231 and 24 hours for MCF7 in transwell invasion assay). Migrated cells **(b) **and invasive cells **(c) **were counted and the results were expressed in comparison to control. (***, *P *< 0.001; student's t-test).**(d, e) **MDA-MB-231 and MCF7 cells were pretreated with 5 nM staurosporine, a PKC inhibitor, for 3 hours and then along with N-HDL and H-HDL at 100 μg/ml apoA-I respectively for another 6 hours. Cell adhesion to HUVEC **(d) **and attachment to ECM **(e) **were measured. (***, *P *< 0.001; student's t-test).

It has been reported that the activity of PKC plays a vital role in regulating tumor development including cell migration, invasion and adhesion and etc. Therefore, the ability of N-HDL and H-HDL to induce PKC activity was examined. It was found that H-HDL stimulated higher PKC activity compared with N-HDL both in MDA-MB-231 and MCF7 cells (Figure 4A). These results suggest that H-HDL may promote breast cancer cell metastasis via the PKC pathway. In order to test this, MDA-MB-231 and MCF7 cells were treated with staurosporine, a PKC inhibitor, in order to determine the role of PKC pathway in the cell migration, invasion and adhesion promoted by H-HDL. Staurosporine is a powerful inhibitor of breast cancer cell migration (Figure 4B), invasion (Figure 4C), adhesion to HUVEC (Figure 4D) and to ECM (Figure 4E). After pretreatment with staurosporine, the H-HDL stimulated MDA-MB-231 and MCF7 cell migration, invasion, adhesion to both HUVEC and ECM was substantially reduced, and no statistical significance was found among the groups of staurosporine-pretreated cells in all the above assays.

### siRNA targeted at PKC significantly reduces hypochlorite-modified HDL-induced invasion, adhesion and integrins expression of MDA-MB-231 cells

**Figure 5:**
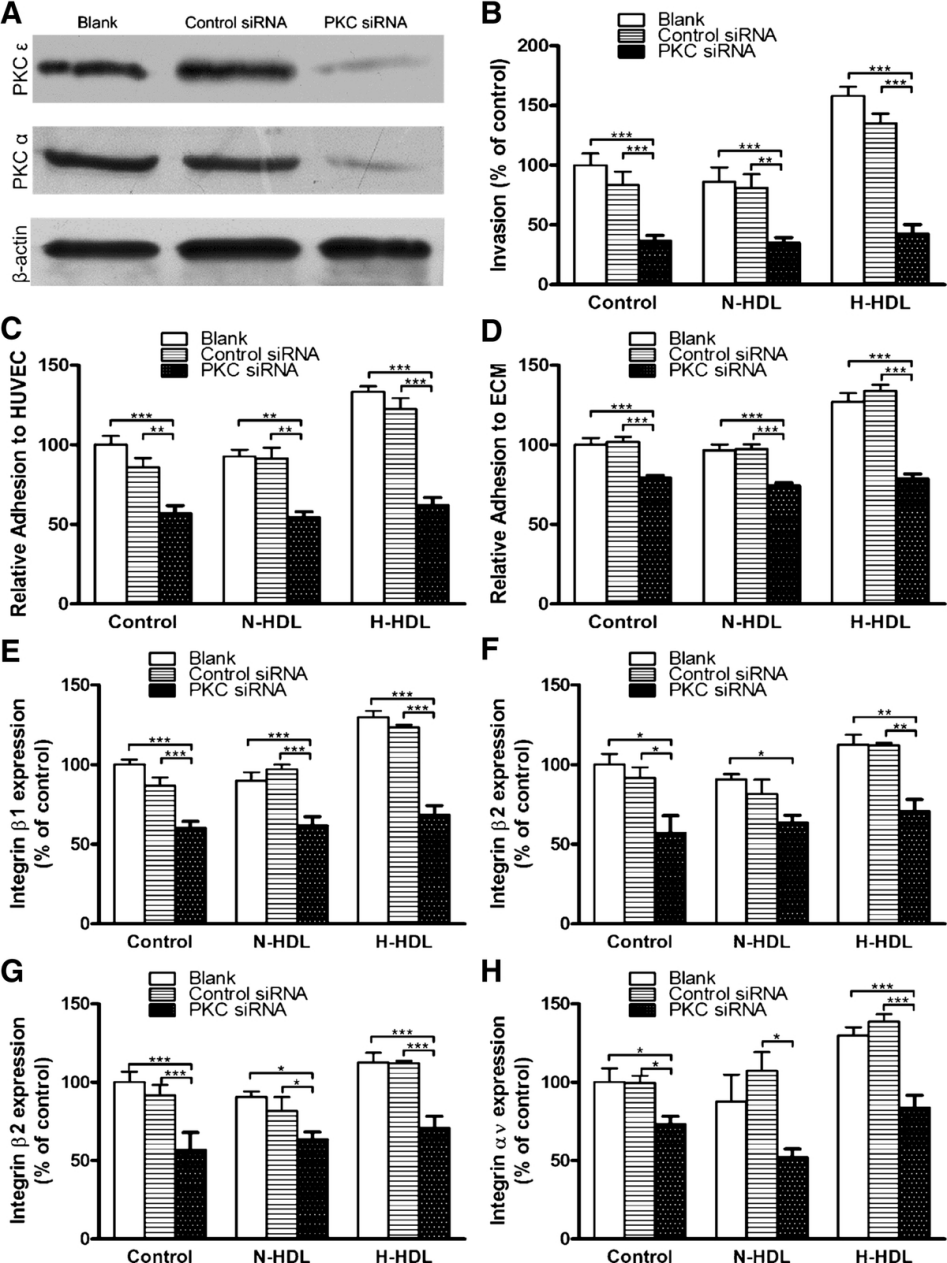
**The effect of silencing PKC on the invasion, adhesion and integrins expression of MDA-MB-231breast cancer cells**. MDA-MB-231 cells were left untreated or transfected with scramble siRNA (control siRNA) or siRNA for PKC (PKC siRNA), and then were incubated with N-HDL and H-HDL respectively for indicated hours as previously described. The effect of control siRNA and PKC siRNA on the protein expression of PKC subtypes was determined by western blot **(a)**. Cell invasion **(b)**, adhesion to HUVEC **(c)**, attachment to ECM **(d)**, and integrin levels **(e, f, g, h) **on the cell surface were examined. (*, *P *< 0.05; **, *P *< 0.01; ***, *P *< 0.001; one-way ANOVA).

PKC siRNA was applied further to investigate the role of PKC in the H-HDL induced invasion, adhesion and integrins expression of breast cancer cell. The effect of PKC siRNA on the cellular PKC expression was shown (Figure 5A). H-HDL induced MDA-MB-231 cell invasion (Figure 5B), adhesion to HUVEC (Figure 5C) and attachment to ECM (Figure 5D) was dramatically reduced, and no statistical significance was found among the groups of PKC siRNA cells in all the above assays. In addition, MDA-MBV-231 cells transfected with PKC siRNA showed a substantial decrease in the integrins expression even after treated with H-HDL (Figure 5E, F, G, H). These results taken together suggests that the increased abilities of H-HDL to promote MDA-MB-231 and MCF7 cell migration, invasion, and adhesion to HUVEC and ECM is mainly owing to the increased level of PKC.

### Hypochlorite-modified HDL promotes breast cancer cell metastasis *in vivo*

**Figure 6:**
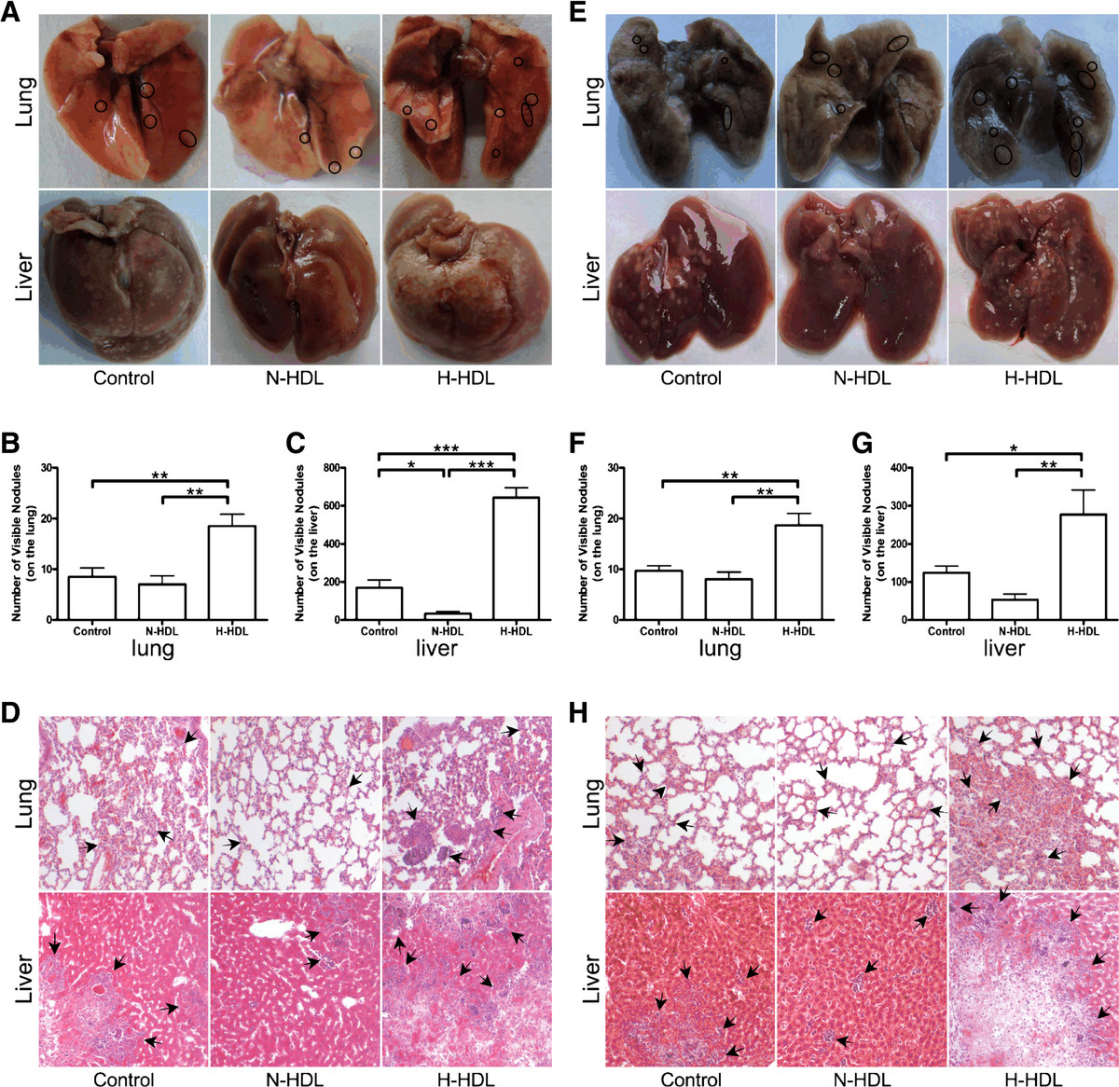
**H-HDL promotes pulmonary and hepatic metastasis of MDA-MB-231 and MCF7 cells *in vivo***. **(a) **MDA-MB-231 and MCF7 cells were pretreated with N-HDL and H-HDL for 24 hours then 4 × 10^5 ^cells were intravenously injected into BALB/c nude mice via tail vein. After 20 days, lungs and livers were resected and analyzed for metastasis. Representative pictures of lungs and livers were shown. **(b, c) **Quantitative evaluation of detectable nodules on the surface of the whole lungs and livers was shown. Data is expressed as mean ± SEM. (*, *P *< 0.05; **, *P *< 0.01; ***, *P *< 0.001; one-way ANOVA). **(d) **Representative histological photomicrographs of lung and liver tissue sections stained with H&E (10×). Arrows indicate tumor islands. **(e) **MDA-MB-231 and MCF7 cells were pretreated with N-HDL and H-HDL for 24 hours then 2 × 10^6 ^cells were injected into the mammary fat pad. BALB/c nude mice were received normal or oxidized HDL injection through vein tail every three days. After 40 days, lungs and livers were resected and analyzed for metastasis. Representative pictures of lungs and livers were shown. **(f, g) **Quantitative evaluation of detectable nodules on the surface of the whole lungs and livers was shown. Data is expressed as mean ± SEM. (*, *P *< 0.05; **, *P *< 0.01; one-way ANOVA). **(h) **Representative histological photomicrographs of lung and liver tissue sections stained with H&E (10×). Arrows indicate tumor islands.

The above data indicates that N-HDL has an elevated ability to promote breast cancer cells proliferation, migration, invasion and adhesion to both HUVEC and ECM compared with N-HDL *in vitro*. In order to determine if these effects also occur *in vivo*, we utilized a tail vein metastasis model and a mammary fat pad spontaneous metastasis model to check the metastasis ability of HDL-treated MCF7 cells in nude mice (Figure 6A). For the tail vein metastasis model, H-HDL significantly promoted both pulmonary and hepatic metastasis of MCF7 cells as was observed by an increase in the nodules on the lungs of H-HDL group by 164.3% and 117.6% as compared with that of N-HDL and control (both at *P *< 0.01; Figure 6B). In addition, the nodules on the livers of H-HDL group increased by 1840% and 281.6% compared with that of N-HDL and control (both at *P *< 0.001; Figure 6C). On the contrary, N-HDL reduced metastasis of MCF7 cells in the liver by 80.3% as compared with control (*P *< 0.05; Figure 6C). The results from the mammary fat pad spontaneous metastasis model are very similar. The data indicated that H-HDL significantly promote both lung and liver homing of MDA-MB-231 cells (Figure 6E). The nodules on the lungs of H-HDL group increased by 133.4% and 93.1% (both at *P *< 0.01; Figure 6F) and the nodules on the livers of H-HDL group increased by 420% and 123.4% compared with that of N-HDL and control (*P *< 0.01 for N-HDL and *P *< 0.05 for control; Figure 6G). Representative histological photomicrographs of lung and liver tissue sections stained with H&E (Figure 6D, H).

## Discussion

Previous studies suggest that breast cancer patients are exposed to conditions of oxidative stress [31,32]. Oxidative stress is caused by excess generation of reactive oxygen species (ROS) and/or decreased antioxidant level in the target cells and tissues and is closely related to all aspects of cancer, from carcinogenesis treatment and prevention [33]. ROS are capable of altering all major classes of biomolecules, such as lipids, nucleic acids and proteins. These changes may not only alter the structure of these molecules but may also change the function [34]. Furthermore, there is a growing evidence for an involvement of lipids, lipid metabolism and lipid peroxidation in breast cancer development [35-37]. Lipid alterations and the susceptibility of lipoproteins to oxidation may play important roles in breast cancer. Several studies have demonstrated that there are significant changes in serum lipids and lipoproteins in cancer patients. These changes include elevated plasma lipid level such as total lipids, triglycerides, total cholesterol, LDL and free fatty acids (FFA) along with low concentrations of HDL [38,39]. Despite these observations, the relationship between oxidative stress and breast cancer is still not clear. The purpose of this report is to provide a study of the effect of hypochlorite-oxidized HDL on the promotion of breast cancer development and to demonstrate the significantly different effects between hypochlorite-oxidized HDL and normal HDL on breast cancer cell metastasis progression.

HDL plays an extremely important role in protecting the cardiovascular system from atherosclerosis. This includes HDL's roles in reverse cholesterol transport (RCT), along with HDL's anti-oxidant, anti-inflammatory, anti-thrombotic properties [15,16]. Of note, HDL can exert its anti-oxidant activity by inhibiting generation of reactive oxygen species (ROS) both *in vitro *[40,41] and *in vivo *[42]. The antioxidative activity of HDL is related to the presence of several apolipoproteins (apoA-I, apoE, apoJ, apoA-II and apoA-IV) and enzymes (PON1, PAF-AH, LCAT, GSPx) with antioxidative properties.

However, HDL was found to be modified in many ways under certain disease states and these modifications can impair its functions. We previously found that HDL in type 2 diabetes mellitus can be modified, and such modified HDL was found to promote breast cancer cell metastasis *in vivo *and increase cell proliferation, migration, invasion and adhesion s *in vitro *as compared with normal HDL [23,24]. Similar to type 2 diabetes, oxidative stress has been reported to be important in breast cancer. This taken together with the susceptivity of HDL to be modified under conditions of oxidative stress, drove us to speculate that oxidized HDL may have an altered function in development and progression of breast cancer.

In our present study, we have utilized both an *in vitro *model system, using both hormone-independent (MDA-MB-231) and hormone-dependent (MCF7) cell lines, and an *in vivo *model utilizing both a metastasis model and a spontaneous metastasis model. The *in vitro *experiments demonstrate that hypochlorite-oxidized HDL can promote cell migration, invasion and adhesion to HUVEC and ECM. These studies also indicate that hypochlorite-oxidized HDL can induce higher cell surface expression of integrins and increased activity of PKC. Moreover, we observed that antibodies against integrins, PKC inhibitor (staurosporine), and siRNA targeted at PKC can inhibit hypochlorite-oxidized HDL induced breast cancer cell invasion and adhesion to HUVEC and ECM. In addition, integrins expression was regulated by PKC. The present data demonstrates that the increased capabilities of hypochlorite-oxidized HDL in promoting breast cancer cell invasion and adhesion to HUVEC and ECM is mainly due to the elevated PKC activity.

The *in vivo *models used in this study include a tail-vein injection model utilizing MCF7 cells and a spontaneous metastatic model utilizing MDA-MB-231 cells. These experiments clearly show that hypochlorite-oxidized HDL compared with normal HDL, can promote breast cancer cells metastasis *in vivo*. The experimental results from the *in vitro *and *in vivo *experiments allows us to speculate that the promoting effects of HDL under oxidative stress on breast cancer cell metastasis, at least partially, may be attributed to the oxidation of HDL.

The potent oxidant hypochlorite is a highly aggressive ROS and is capable of inducing lipid peroxidation and protein oxidation. Importantly, both lipid peroxidation and protein oxidation in HDL could have potential roles in neoplastic transformation and development. Due to the complicated composition of HDL, further intensive research needs to be focused on the determination of the oxidatively modified parts of HDL that is responsible for the promotion of breast cancer development.

## Conclusion

Our study demonstrates that the modifications of HDL that may occur in breast cancer patients could lead to accelerated breast cancer metastasis and progression. These results suggest that an additional avenue for the pursuit of therapeutic strategies for patients with breast cancer may be based on the adverse effects of oxidized HDL on breast cancer metastasis.

## Abbreviations

HDL: high-density lipoprotein; LDL: low-density lipoprotein; apoA-I: apolipoprotein A-I; TC: tumor cells; HUVEC: human umbilical vein endothelial cells; ECM: extracellular matrix; PKC: protein kinase C

## Competing interests

The authors declare that they have no competing interests.

## Authors' contributions

BP, HR and BY performed all cell function experiments. BP, HR and JK performed the mouse experiments. YM, FY and CN carried out the isolation and hypochlorite-oxidation of HDL. BP, XL, YH, YZ and LZ conceived and designed the experiments and analyzed the data. The manuscript was written by BP, HR, BW and YZ. All authors read and approved the final manuscript.
